# Assessment of Human Sleep Depth Is Being De-Standardized by Recently Advised EEG Electrode Locations

**DOI:** 10.1371/journal.pone.0071234

**Published:** 2013-08-05

**Authors:** Bob Kemp, Paul van Someren, Marco Roessen, J. Gert van Dijk

**Affiliations:** 1 Department of Neurology, Leiden University Medical Centre, Leiden, The Netherlands; 2 Sleep Centre, Medical Centre Haaglanden, Den Haag, The Netherlands; University of California, Riverside, United States of America

## Abstract

Human sleep depth was traditionally assessed by scoring electro-encephalographic slow-wave amplitudes at the globally standardized C4-M1 electrode derivation. Since 2007, the American Association of Sleep Medicine (AASM) has accepted three additional derivations for the same purpose. These might well differ in slow wave amplitudes which would bias the scorings. Some derivations might also introduce large inter-individual variability. We compared mean and variability of slow wave amplitudes between six derivations including the four AASM ones. Slow wave amplitudes in those derivations were simultaneously measured using automated analysis in 29 patients. Each amplitude was divided by the average from the six derivations, thus removing shared factors such as age, gender and sleep depth while retaining factors that differ between the derivations such as caused by local skull characteristics, electrode distance and neuronal dipole orientation. The remaining inter-individual variability differed significantly and up to a factor of two between the AASM derivations. The amplitudes differed significantly and up to 60% between the AASM derivations, causing substantial scoring bias between centres using different derivations. The resulting de-standardization most likely affects any patient group because the amplitude differences were consistent over diagnoses, genders, and age. Derivation-dependent amplitude thresholds were proposed to reduce the scoring bias. However, it would be better to settle on just one derivation, for instance Cz-Oz or Fpz-Cz because these have lowest variability while matching the traditional C4-M1 amplitudes.

## Introduction

Analysis of human sleep depth is mainly determined by the amplitude of slow waves in the scalp electro-encephalogram (EEG). The now globally applied sleep scoring manual of the American Association of Sleep Medicine (AASM) defined EEG-electrode pairs (derivations) F4-M1 or Fz-Cz for the measurement of slow waves [1]. Although the former is called ‘recommended’ and the latter ‘alternative’, item G.1. of AASM's frequently-asked-questions list (FAQ) stated both derivations to be equally acceptable [2]. However, item V.5. of that FAQ stated that the Fz-Cz derivation may suffer from ‘slow-wave cancellation’ in some subjects, meaning that the Fz-Cz amplitude in those subjects is relatively small because their neuronal slow-wave dipole is oriented in such a way that the Fz and Cz locations receive about equal potentials. As the dipole orientation can vary between subjects this may cause variation in slow-wave amplitude, independent from sleep depth. To avoid this, the AASM suggested two other derivations, Fpz-E1 and C4-M1 [2], [3]. As a result the once-universal traditional standard derivation (C4-M1, see [4]) has been supplanted by a range of choices. Different sleep centres may prefer different derivations. In that case, their scorings may become mutually incompatible if slow-wave amplitudes differ systematically between derivations.

Any systematic amplitude difference between derivations can be compensated by applying a specific amplitude threshold for each derivation that will result in the same amount of slow wave sleep. This will not solve all problems however: it is possible that the inter-individual variability of slow wave sleep amplitude differs systematically between derivations. If so, this source or error can only be minimized by selecting an optimal derivation, i.e. the one with the smallest variability. The present contribution compared variability in slow wave amplitude between derivations and reports thresholds that correct any systematic bias. The results from this comparison can be used by standardization bodies to re-standardize the procedure for the assessment of sleep depth.

## Materials and Methods

A simple method to compare inter-individual variability of slow-wave amplitude between derivations is described below. This variability as well as the mean amplitude were analyzed for each AASM derivation. The Cz-Oz and Fpz-Cz derivations were added to the analysis because both have amplitudes not very different from the C4-M1 EEG and the latter one also yields similar sleep scores [5].

### Patients and recorded data

In order to adapt to the common practice as recommended by the AASM, the Leiden department of Neurology added the F4 electrode to all ambulatory sleep recordings of patients not involved in any research protocol. The recordings obtained in this way during the first year were used for this study. Ethics approval and patient consent were not necessary because data were gathered exclusively in the context of common-practice patient care. Dutch law does not require approval or consent in this case. Our present research on that data was done later and did not subject people to any other treatment and did not prescribe any other behaviour to people. Because of that, section “b” of Article 1 of Paragraph 1 of the Dutch law about “Medical-scientific research on people” (the law about Ethics requirements) makes clear that the law does not apply to this case [6]. Normal regulations regarding privacy and confidentiality apply. The patient data that we used is anonimized and can in no way be related to the patient. We carried out the research with the highest esteem to patients' privacy and rights. Because of that esteem, we did not make their data publicly available. All patient data is kept at the Leiden department of neurology during fifteen years after the date of the investigation. Article 467 of Civil Law Book 7, Title 7, Section 5 (the section about medical treatment of patients) explicitly states that the way we retrospectively used patient data is considered fully legal and just in The Netherlands [7]. Patients did not raise any objection as mentioned in this article.

**Figure 1:**
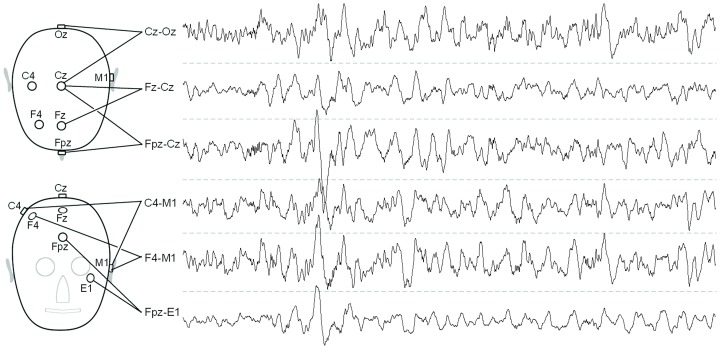
Compared EEG derivations. Locations of electrodes involved in the studied derivations (left) with 30 seconds of EEG recorded in +/−100 µV panes (right). Top: top view of the head (nose pointing downwards) with Cz-Oz, Fz-Cz and Fpz-Cz EEG derivations. Bottom: front view of the head with C4-M1, F4-M1 and Fpz-E1 EEG derivations. Note the difference in average amplitude between the derivations. Note also that the large waves at 7 and 23 seconds peak at different locations.

An Embla Titanium recorder simultaneously recorded EEG's from F4, C4, Fpz, Fz, Cz, Oz, E1 and M1 electrodes, each one referred to the Pz electrode. From these eight EEG's, the F4-M1, Fz-Cz, Fpz-E1, C4-M1, Cz-Oz and Fpz-Cz EEG derivations (Fig. 1) were constructed off-line by subtraction. The frequency bandwidth was 0.15–120 Hz and the EEG was digitized at 256 Hz. Sleep stages were scored according to version 1 of the AASM manual [1]. All recordings were included that showed at least two periods of deep NonREM sleep (stage N3, based on C4-M1), each period lasting at least five minutes and being free of artifacts or intrusion from other stages. In order to possibly capture some variations caused by sleep physiology and also to insure that no overlapping periods could be selected, the two periods had to be separated by REM sleep.

**Figure 2:**
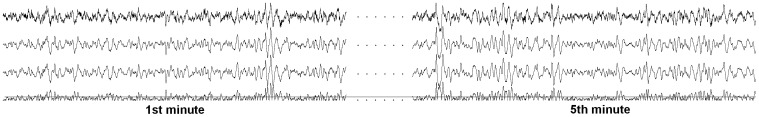
Computation of slow-wave amplitude, SWA. First and last minute of a typical five-minute period of N3 sleep. Traces from top to bottom show the C4-M1 EEG unfiltered (trace 1) and after bandpass filtering (trace 2) and additional Notch-filtering (trace 3) and finally also rectification (trace 4). The dotted lines indicate the 0 µV level and the vertical distance between those lines is 200 µV. The solid horizontal line in trace 4 shows its 5-minute average, SWA, which in this case was 24 µV.

Each period was selected using Polyman review software (www.edfplus.info > downloads). This software computed for each derivation the mean slow-wave amplitude (SWA) in each period as follows (Fig. 2). First, the EEG was band-pass filtered (second-order with 3 dB cut-off frequencies at 0,5–2 Hz), notch filtered (at 50 Hz with 1 Hz bandwidth), and rectified. The result was then averaged over the five-minute period, giving one SWA value for that period. This automatic method was preferred over any human assessment of slow-wave amplitudes because the latter might introduce subjective variability between derivations. Mean peak-to-peak amplitudes in manual sleep scoring are directly proportional to SWA values as computed here for any, even non-Gaussian, distribution. In fact, most automatic methods that detect N3 sleep are based on average slow-wave amplitude (or its square, slow-wave power) and closely agree with human scoring of N3 sleep [8], indicating that thresholding SWA mimics the human method of comparing each slow-wave to a threshold. This implies that comparing SWA values between derivations corresponds to comparing visually assessed peak-to-peak amplitudes but without scorer bias and variability.

As six derivations were recorded, six pre-REM and six post-REM SWA values were obtained in each patient. SWA values expressed in µV were automatically stored in a spreadsheet. Data were checked for inconsistencies and outliers. The pre- and post-REM periods were analyzed separately to check consistency of the results.

### Data transformation and statistical analysis

**Figure 3:**
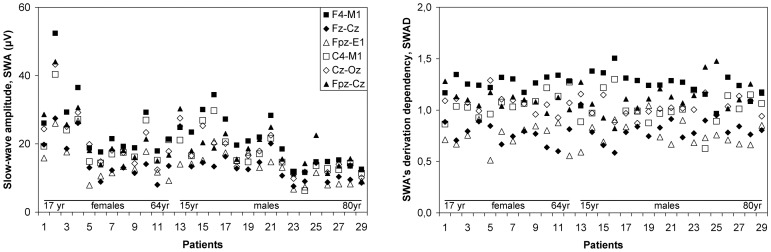
Inter-individual slow-wave variability, per derivation. Mean slow-wave amplitude (SWA, left graph) and its derivation-dependency (SWAD, right graph) of 29 patients (sorted by age and gender on the horizontal axis) for each derivation (legend in the left graph), in the first 5-minute period. Note that subjects generating large SWA's (mostly young women as opposed to old men) tend to do so in all derivations. Note also that SWAD does not show these effects, but does retain the differences between derivations.

The effect of a derivation on the inter-individual mean and variability of SWA could be obscured by factors such as age and gender (Fig. 3, left) and of course also by sleep depth. Those factors are common to all derivations and were removed by dividing the SWA value of a specific derivation by the average of all six derivations in the same period and patient. The thus transformed variable, SWAD (short for SWA's derivation dependency), represents the effects on SWA that differ between the six derivations, such as caused by local skull characteristics, electrode distance and dipole orientation. We will analyze SWAD to quantity the influence of a derivation on SWA.

Whether SWA and SWAD were normally distributed was tested with the Shapiro-Wilk test. The group mean of SWAD was computed for each derivation in order to quantify the systematic effect of that derivation on slow-wave amplitude. The inter-individual coefficient of variation, CV, of SWAD was computed for each derivation in order to compare SWA variability between derivations; the CV is the standard deviation divided by the mean. Mean and CV were also computed using SWA instead of SWAD, in order to assess to what extent the transformation affected the results.

One-way repeated measures ANOVA assessed whether SWAD differed significantly between derivations. ANOVA included multiple t-tests with Bonferroni adjustment of the 0.05 significance level to assess which pairs of derivations caused the difference. A correction factor to normalize the slow-wave amplitude of a particular derivation to that of the C4-M1 derivation was computed by dividing the group mean of SWAD at that derivation by the group mean at C4-M1. Since the transformation also removed any effect of sleep depth, no difference was expected between the first and second five-minute period. The precision of the six correction factors and of the six CV's was therefore assessed by analyzing the difference between those two periods, using a paired t-test. Correction factors were also computed for subgroups based on diagnosis, gender, and age in order to explore any effects of those.

Statistical analyses were performed using SPSS 17.0. Differences were assessed at the two-sided 5% significance level. Since the derivations were measured simultaneously, SPSS offers no test for a mutual comparison of their CV's (Levene's test for comparing variances can only be applied to independent samples). Instead, we tested whether the CV's differed by at least two confidence intervals.

## Results

The six EEG derivations were simultaneously recorded in 95 patients suffering from various sleep disorders. Many patients did not show the required two artifact-free periods of N3 sleep because they had insufficient N3 sleep or too many artifacts. After excluding such cases 29 patients were left. Diagnoses were PMLS (n = 3), OSAS (n = 7), narcolepsy (n = 8) and idiopathic hypersomnia (n = 10), while one patient suffered from both narcolepsy and OSAS. Mean age was 43 (range 15–80) and twelve patients were female.

Fig. 3 shows the mean slow-wave amplitude, SWA, and its derivation-dependency, SWAD, for each patient and derivation, in the first five-minute period. Table 1 summarizes group statistics of SWA and SWAD, for each derivation and in both periods. Group histograms of the twelve SWA's (two periods, six derivations) indicated non-Gaussian distributions, as was confirmed by the Shapiro-Wilk test of normality (eleven p-values<.04, one p = .06). Group histograms of the twelve SWAD's were fairly normal as was confirmed by the Shapiro-Wilk test (two p-values<.04, ten p-values>.05).

**Table 1:** Comparing derivations: results from both five-minute periods.

Period	Derivation	Mean		Mean/(C4-M1)		CV (Max/Min)
		SWA	SWAD (95% c.i.)	SWA	SWAD	SWAD
1	F4-M1	22.5	1.25 (1.21–1.29)	1.22	1.21	0.085 (1.56)
2	F4-M1	22.2	1.24 (1.21–1.27)	1.20	1.19	0.069 (1.49)
1	Fz-Cz	14.1	0.79 (0.75–0.83)	0.76	0.77	0.132 (1.80)
2	Fz-Cz	14.1	0.78 (0.74–0.81)	0.76	0.75	0.117 (1.67)
1	Fpz-E1	13.6	0.77 (0.72–0.81)	0.74	0.74	0.162 (2.03)
2	Fpz-E1	13.9	0.78 (0.73–0.83)	0.75	0.75	0.159 (1.92)
1	C4-M1	18.4	1.03 (0.98–1.09)	1.00	1.00	0.137 (2.08)
2	C4-M1	18.5	1.04 (0.98–1.10)	1.00	1.00	0.145 (2.65)
1	Cz-Oz	18.6	1.04 (1.01–1.08)	1.01	1.01	0.089 (1.48)
2	Cz-Oz	18.5	1.03 (0.99–1.07)	1.00	0.99	0.096 (1.40)
1	Fpz-Cz	19.8	1.12 (1.07–1.17)	1.08	1.08	0.120 (1.80)
2	Fpz-Cz	20.5	1.14 (1.09–1.18)	1.11	1.09	0.111 (1.57)

The first two columns indicate the analyzed five-minute periods and derivations. Subsequent columns show, for each period and derivation, group means of slow-wave amplitude (SWA, in µV) and its derivation-dependent component (SWAD), followed by the same means but now relative to the mean of the C4-M1 derivation, and finally the coefficient of variation (CV) of SWAD. Brackets in the third and last column contain SWAD's 95% confidence interval and Max/Min ratio, respectively.

ANOVA showed that SWAD differed highly significantly (p<0.001) between derivations, in both periods. The corresponding t-tests showed that SWAD of the traditional C4-M1 derivation differed highly significantly (p<0.001) from the F4-M1, Fz-Cz and Fpz-E1 derivations, but not (p>0.8) from the Cz-Oz and Fpz-Cz ones, in both periods.

The correction factor that normalizes a derivation's amplitudes to C4-M1 amplitudes is shown in the fifth column of Table 1. When averaged over the two periods, the correction factors for the significantly different F4-M1, Fz-Cz and Fpz-E1 amplitudes were 1.20, 0.76 and 0.75, respectively. The same computation based on SWA instead of SWAD (fourth column of Table 1) resulted in practically the same correction factors: 1.21, 0.76 and 0.75, respectively. Paired t-tests comparing the correction factors between the first and second period showed that the differences had a standard deviation of 0.014 and a 95% confidence interval of 0.028. Table 2 shows the correction factors (based on SWAD and averaged over the two periods) for several subgroups of patients.

**Table 2:** Amplitude correction factors for various patient groups.

	All	OSAS	Narco	Id.Hyp	Men	Women	<41 yr	>40 yr
	29	7	8	10	17	12	14	15
F4-M1	1,20	1,20	1,19	1,29	1,22	1,18	1,23	1,18
Fz-Cz	0,76	0,78	0,70	0,84	0,80	0,70	0,76	0,75
Fpz-E1	0,75	0,72	0,71	0,82	0,78	0,70	0,74	0,75
C4-M1	1,00	1,00	1,00	1,00	1,00	1,00	1,00	1,00
Cz-Oz	1,00	0,98	0,98	1,10	1,01	0,99	1,02	0,98
Fpz-Cz	1,09	1,07	1,02	1,21	1,14	1,02	1,07	1,11

The first column indicates the derivation. The subsequent 8 columns contain the amplitude correction factor of that derivation (averaged over the two five-minute periods) in various patient groups. Grouptype and groupsize are indicated in the first and second row, respectively. Note that in all groups, F4-M1 has the largest amplitudes while Fz-Cz and Fpz-E1 have the smallest and Cz-Oz is closest to the traditional C4-M1 amplitudes.

The last column of Table 1 shows the coefficient of variation, CV, of SWAD. The same column shows (between brackets) the maximum SWAD among patients divided by the minimum. When averaged over the two periods, the CV's for the F4-M1, Fz-Cz, Fpz-E1, C4-M1, Cz-Oz and Fpz-Cz derivations were 0.077, 0.124, 0.160, 0.141, 0.093 and 0.115, respectively. Paired t-tests comparing those CV's between the first and second period showed that the differences had a standard deviation of 0.011 and a 95% confidence interval of 0.022. The F4-M1 derivation had the smallest CV. It was at least two confidence intervals smaller than Fz-Cz, Fpz-E1 and C4-M1, in both periods. The difference to the two other derivations (Cz-Oz and Fpz-Cz) was less than two confidence intervals, also in both periods. The variability of SWA was much larger than that of SWAD, with CV's of 0.38, 0.41, 0.39, 0.38, 0.39 and 0.39 in the respective derivations.

## Discussion

The most commonly used AASM derivation, F4-M1, had 20% larger amplitudes than the traditional Rechtschaffen and Kales derivation, C4-M1. The use of the F4-M1 derivation will therefore cause amplitudes to cross the 75 µV scoring threshold more often, as is our own experience and was also concluded in another study [9] in which AASM scoring resulted in 9 minutes more deep sleep and 20 minutes less light sleep. That study also showed that this scoring bias differs between individuals: it decreased in subjects with very large amplitudes because the amplitude threshold was then crossed anyhow, in both derivations. This effect probably explains why a study in children that confirmed the decrease of light sleep, only showed a non-significant increase of deep sleep [10]. We found that all four derivations advocated by the AASM differed significantly in amplitude and some differed by even 60%. So, the incompatibility between centres using the latter derivations will be even larger than reported in the Moser study, and differ between individuals. The differences occur consistently and independent of diagnoses, age, and gender (see Table 2), probably because these factors do not substantially affect dipole orientation or other anatomical differences between derivations. The strong consistency between patient groups suggests that dipole orientation is relatively sturdy which would mean that the bias not only occurs in the studied groups but also in others. Obviously, this scoring bias represents a considerable problem of the AASM scoring standard.

The amplitudes of the C4-M1, Fpz-Cz and Cz-Oz derivations did not mutually differ significantly, probably because all have a central electrode (picking up the central end of the slow-wave dipole) and a relatively distant reference electrode (picking up the potential about halfway both ends). The scoring bias of the other derivations can be largely reduced by applying specific thresholds instead of the traditional 75 µV scoring threshold of the C4-M1 derivation. The thresholds are 90, 57 and 56 µV for the F4-M1, Fz-Cz and Fpz-E1 derivations respectively and correct the average amplitude differences between those derivations and C4-M1. In this way, the total time during which slow waves cross these thresholds will be equalized between derivations, which will strongly reduce the scoring bias. However, the various derivations may still bias the scorer due to other EEG waves and artifacts. Bias also occurs in individual cases because the derivations are differently affected by inter-individual anatomical and functional variations, as was demonstrated in the present study. The data from the first and second five-minute period were strikingly equal, indicating that the average slow-wave topography was stable across the night, as was also demonstrated in patients with ADHD [11]. However, the topography may change across the night. In fact, varying contributions of several slow wave generators can dynamically change on a timescale of seconds, as illustrated by Fig. 1, or in the course of the night depending on homeostatic regulation and brain development [12]. So, although the suggested thresholds would reduce the scoring bias between the four AASM derivations, several differences can not be ruled out and it would be better to harmonize scorings by using the same derivation everywhere.

The concern expressed in the AASM-FAQ about inter-individual SWA variability being dependent on the derivation was confirmed by our data. We found the smallest variability in the F4-M1 derivation with a CV of 0.08 but still a maximum difference between subjects of about 50%. Significantly larger CV's were found in the three other AASM derivations. The particular concern about the Fz-Cz derivation was not confirmed: the C4-M1 and Fpz-E1 derivations, introduced because of that concern, had an even larger CV and a maximum difference between subjects of about 100%.

The difference in variability between derivations could only be assessed after removing the effect of factors such as age, gender and sleep depth that have a common effect on all derivations. The resulting amplitudes, SWAD, had CV's ranging from 0.08 till 0.16 while the original amplitudes, SWA, had CV's of 0.38 till 0.41. Apparently the common factors had a relatively large effect on slow-wave variability. This does not relax the need to select a low-CV derivation because most studies control for, or actually study, those common factors. For instance, when comparing the duration of slow-wave sleep of an adult male to normal values for that category, effects of age and gender are controlled for and sleep depth is analyzed, leaving as confounding variable only the inter-individual anatomical variations as represented by SWAD. These variations are relatively large for the Fpz-E1 or C4-M1 derivations. Therefore, normal values of SWA for adult males based on those derivations would require unnecessarily large ranges: larger than those for F4-M1 or Cz-Oz derivations.

The non-AASM derivations Fpz-Cz and Cz-Oz are attractive alternatives because their CV's and amplitudes did not differ significantly from either the low F4-M1 variabilities or the traditional C4-M1 amplitudes.

The AASM proposal to replace the traditional C4-M1 derivation to measure slow waves by F4-M1 and Fz-Cz derivations was hardly based on quantitative data. Therefore no thresholds were specified to supplant the traditional 75 µV one, even though F4-M1 amplitudes now appear to be about 60% higher than Fz-Cz ones. This lack of data also explains why the alleged ‘cancellation’ problem of Fz-Cz could lead to the acceptance of two other derivations, Fpz-E1 and C4-M1, that now appear to even aggravate the problem. Finally, the Fpz-Cz and Cz-Oz derivations that actually do reduce the impact of the problem, while retaining compatibility to the 75 µV threshold, were not considered by the AASM. These examples support earlier and more general comments [13] that evidence such as presented here should underlie any dismissal or modification of a widely used standard.

Our results show that bias occurs between sleep centres using different slow-wave EEG derivations, which implies that the AASM scoring rules have actually resulted in de-standardization. The differences were consistent and large, so studies in other patient groups and based on manual scoring will most likely confirm this finding, be it with possibly somewhat different correction factors and variabilities than the ones we arrived at. We hope that our results will cause awareness of the problem so that it will be dealt with, either using principles outlined here to correct for amplitude differences or by settling on just one derivation.

Of the studied derivations, Cz-Oz seems best suited for the scoring of NonREM sleep because its amplitudes are closest to the traditional C4-M1 amplitudes while its variability is about as low as the F4-M1 variability. However, selecting the optimal slow-wave derivation does not only depend on those parameters. For instance, the Fpz-Cz derivation is favored by our technicians because it clearly shows sleep spindles and sawtooth waves and does not suffer from the electrode movements on M1, while its sensitivity to eye blinks and frontalis muscle activity supports rather than disturbs visual analysis. Also, computer analysis is increasingly recognized as a viable addition to sleep scoring [14] and introduces its own particular sensitivities to artifacts. Of those, the inevitable movements of the M1 electrode in F4-M1 and C4-M1 disturb most analyses methods. Of the other derivations, our neuro-loop analysis [15] prefers Cz-Oz for slow-wave analysis because it has no eye movements either, while it prefers Fpz-Cz for spindle analysis because its spindles are bigger and well distinguished from eye movements and muscle activity. In general, data on the effects of artifacts on manual and computer analysis would enable a more comprehensive comparison.
